# Giant Galápagos tortoises; molecular genetic analyses identify a trans-island hybrid in a repatriation program of an endangered taxon

**DOI:** 10.1186/1472-6785-7-2

**Published:** 2007-02-15

**Authors:** Michel C Milinkovitch, Daniel Monteyne, Michael Russello, James P Gibbs, Howard L Snell, Washington Tapia, Cruz Marquez, Adalgisa Caccone, Jeffrey R Powell

**Affiliations:** 1 Laboratory of Evolutionary Genetics, Institute for Molecular Biology & Medicine, Université Libre de Bruxelles, Rue Jeener & Brachet 12, 6041 Gosselies, Belgium; 2 Department of Ecology & Evolutionary Biology, Yale Institute for Biospherics Studies ECOSAVE, Yale University, New Haven, CT 06520-8106, USA; 3 College of Environmental Science and Forestry, State University of New York, Syracuse, NY 13210, USA; 4 Department of Biology, University of New Mexico, Albuquerque, NM 87131, USA; 5 Galápagos National Park Service, Puerto Ayora, Galápagos Islands, Ecuador; 6 Charles Darwin Foundation, Puerto Ayora, Galápagos Islands, Ecuador

## Abstract

**Background:**

Giant Galápagos tortoises on the island of Española have been the focus of an intensive captive breeding-repatriation programme for over 35 years that saved the taxon from extinction. However, analysis of 118 samples from released individuals indicated that the bias sex ratio and large variance in reproductive success among the 15 breeders has severely reduced the effective population size (*N*_*e*_).

**Results:**

We report here that an analysis of an additional 473 captive-bred tortoises released back to the island reveals an individual (E1465) that exhibits nuclear microsatellite alleles not found in any of the 15 breeders. Statistical analyses incorporating genotypes of 304 field-sampled individuals from all populations on the major islands indicate that E1465 is most probably a hybrid between an Española female tortoise and a male from the island of Pinzón, likely present on Española due to human transport.

**Conclusion:**

Removal of E1465 as well as its father and possible (half-)siblings is warranted to prevent further contamination within this taxon of particular conservation significance. Despite this detected single contamination, it is highly noteworthy to emphasize the success of this repatriation program conducted over nearly 40 years and involving release of over 2000 captive-bred tortoises that now reproduce *in situ*. The incorporation of molecular genetic analysis of the program is providing guidance that will aid in monitoring the genetic integrity of this ambitious effort to restore a unique linage of a spectacular animal.

## Background

Conservation genetics is a relatively new, yet growing field of research that can have immediate impact on practical issues confronting efforts to conserve biodiversity. Technical developments of molecular procedures directly applicable to genetic analysis of virtually any organism have made important contributions to these efforts. Here we present a unique example of the use of molecular conservation genetics to detect genetic contamination of an otherwise successful captive breeding-repatriation program of a once critically endangered species.

The genetically distinct population of giant Galápagos tortoises (*Geochelone hoodensis*, at times considered a subspecies of *G. nigra*) occupying the island of Española in the extreme southeastern region of the Galápagos archipelago was in grave danger of extinction in the late 1960s due to hunting activities from sealing, whaling, and pirate ships [1], as well as habitat destruction by feral goats. In response, the Charles Darwin Research Station (CDRS) and Galápagos National Park (PNG) initiated in late 1960s a captive-breeding and reintroduction program [2-4]. By 1965, thorough searches across the island found only 14 remaining individuals (two males and 12 females) and all were transferred to the Breeding Centre on the island of Santa Cruz. In 1977, a third Española adult male from the San Diego Zoo (USA) was incorporated into the breeding program. The first tortoises whose parents originated from Española hatched at CDRS in 1971 and were subsequently released in 1975 after completion of a goat eradication campaign. To date, more than 2000 offspring have been repatriated, the island has undergone significant ecological recuperation, repatriated tortoises now reproduce *in situ*, and the core population of 15 parents continues to reproduce in captivity at the Breeding Centre.

As minimization of inbreeding is probably as important a determinant as rehabilitation of habitat for long-term survival of the repatriated population [5-8], we previously used molecular data to assess how much of the genetic diversity of the 15 breeders is represented in the repatriated population [9]. Analysis of 15 informative microsatellite loci allowed us to determine the captive parents of 118 surviving released individuals collected in 1994; at that time, this represented about 40% of the repatriated population. This analysis indicated that contributions of the 15 breeders are highly skewed. The large variance in reproductive success [10] in combination with a severely female-biased sex ratio [11] reduces the effective population size (*N*_*e*_) to an alarming low value of 5.7 [9]. Because the long-term *N*_*e *_is the harmonic mean across generations [11], we suggested rectifying the low *N*_*e *_in the parental population prior to continuation of the repatriation program. Indeed, estimating that *N*_*e *_for the current repatriated population is 1200, *N*_*e *_over the two generations would only reach 11.3. This is an overestimate as it is based on a 60% survival rate of released offspring (CDRS, personal communication), and on the optimistic assumption that sex ratio and reproductive success are not biased on the island. In other words, the current orchestration of the breeding program generated inbreeding that reduced genetic variation of the current population of about 1200 individuals to a level equivalent to that expected for, at best, a population of 11 unrelated individuals. Inbreeding could be effectively reduced by modification of the breeding program to more nearly equalize reproductive success among breeders.

In order to fine-tune the estimate of *N*_*e *_and analyse its evolution through time, we collected in 2003 and 2004 blood samples from 473 additional tortoises of which 316 were sampled on Española and 157 were samples of F1 individuals awaiting release. The latter point is of importance as it allows us to distinguish between differential reproductive success and differential survival rates [9].

## Results

**Figure 1:**
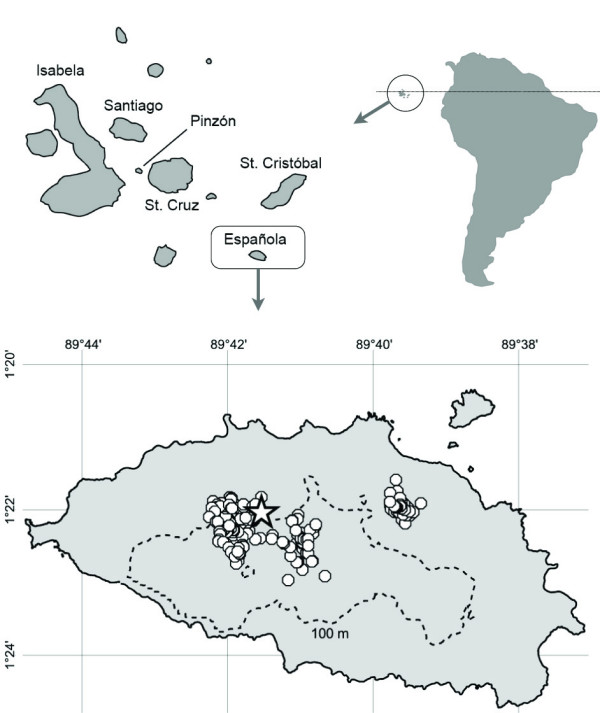
Position of the 316 tortoises blood-sampled on Española in 2003. Circles represent individuals. The star corresponds to the position of individual E1465. The contour dotted-line indicates the 100 meter altitude level. The concentration of sampled individuals in two main areas reflects the localization of suitable habitat where the vast majority of tortoises congregate.

**Figure 2:**
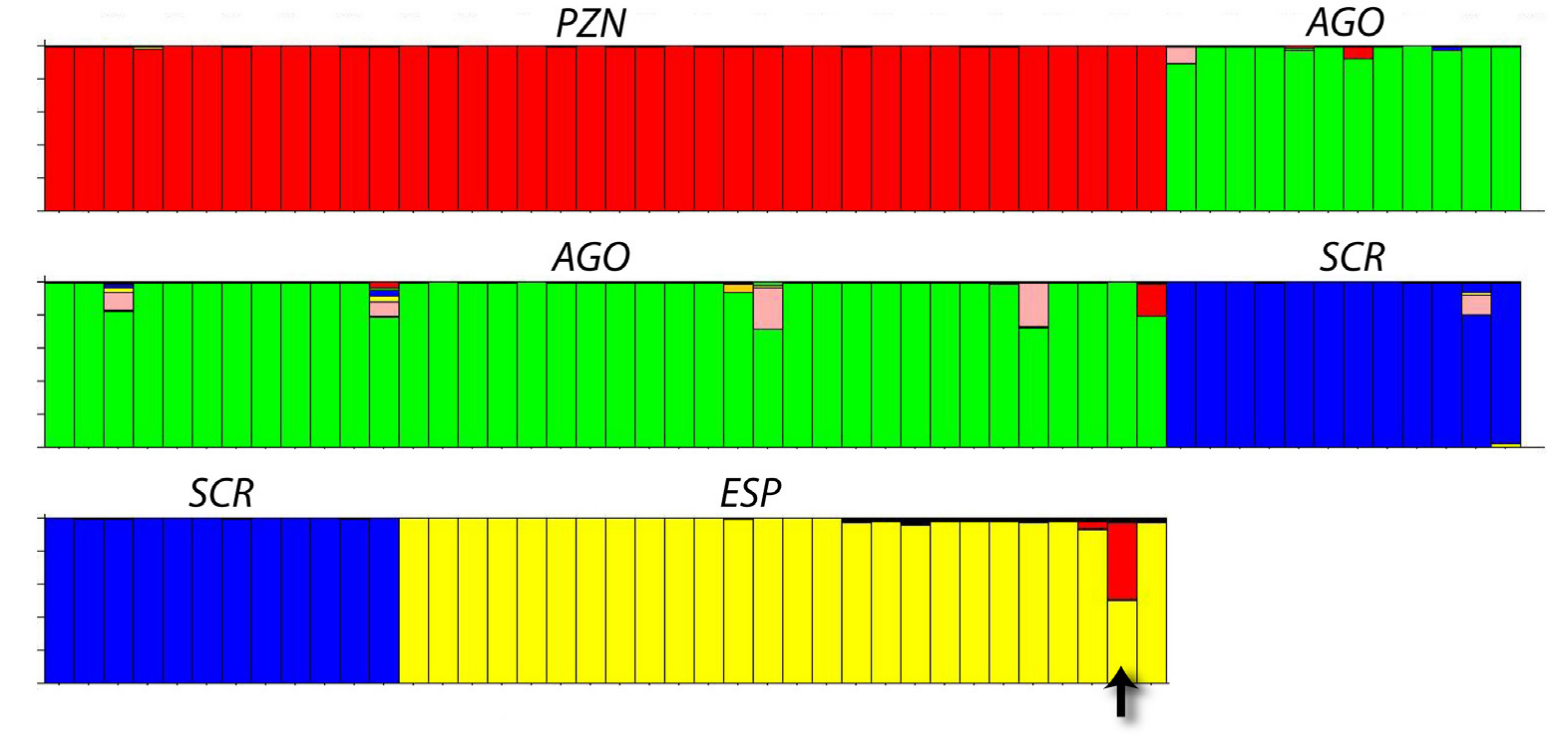
Inferred ancestry of individuals from the islands of Pinzón (*PZN*), Santiago (*AGO*), Santa Cruz (*SCR*), and Española (*ESP*) according to the Structure [17] analysis using prior population definitions. Membership coefficients are colour-coded according to islands: red (Pinzón), green (Santiago), blue (Santa Cruz), and yellow (Española). E1465 (arrow) exhibits membership coefficients of 0.501 and 0.461 for Española and Pinzón, respectively (these values do not add up to one due to nominal associations with other clusters). Structure [17] analysis using a model of population admixture using all available genotypic data from all populations (not just 4) yielded very similar results (data not shown).

Surprisingly, one individual (E1465) sampled on Española (Fig. 1) exhibits one alien allele (*i.e*, not found in any of the 15 captive parents) at 8 of the 15 loci investigated (Table 1). It is therefore impossible that both parents of this individual are among the 15 breeders. Sequencing of a section of the control region in individual E1465 reveals the single haplotype identified previously [12] as specific to the Española population. These results suggest that E1465 is a hybrid between a female Española tortoise and a male from another island. To assess the geographic origin of the father, we genotyped E1465 at 9 loci (of which 4 overlap with the set of loci discussed above) that had been previously used for analysing genetic variability and population structure of giant tortoises across the whole archipelago [13]. The "alien" alleles revealed for E1465 are found at varying frequencies in populations throughout Isabela, Santiago, Pinzón, Santa Cruz, and San Cristóbal. Using a genotypic database of 304 field-sampled individuals from all populations on the major islands, we performed assignment tests using Rannala and Mountain's approach [14] (as implemented [15] in GENECLASS2) and Pritchard et al.'s method [16] (as implemented [17] in STRUCTURE 2.1). The first method assigned E1465 with high probabilities to the Española and Pinzón island populations with log Likelihood values (logL) of -21.839 and -23.169, respectively. All other individuals sampled on Española exhibit much higher logL values (ranging from -4.621 to -10.638; mean -6.965), assigning them to Española. The second approach also indicates that E1465 exhibits a mixed history, with large contributions from both Española and Pinzón (Fig. 2).

**Table 1:** Genotypes at 15 loci for the 15 parents (E1 to E15) from the captive breeding program and for the field-sampled individual E1465

Individual	AC039		AC045		AC063		AC075		AC100		AC111		AC127		AC149		AC190		AC247		AC251		AC263		T45		T68		T70	
																														
E1465	95	99	112	112	80	80	103	115	105	110	133	133	106	110	110	119	108	110	86	130	102	109	95	99	80	86	75	75	86	90
																														
E1 (4)	95	97	100	112	100	100	101	103	105	105	96	96	114	147	117	119	106	108	86	86	102	102	84	112	76	86	75	75	90	90
E2 (2)	97	99	112	112	80	80	101	101	105	120	88	133	97	114	110	110	106	108	86	86	102	118	95	112	86	86	75	85	86	90
E3 (6) *	97	99	100	100	80	100	101	101	105	120	96	133	114	147	119	119	106	108	86	86	102	102	84	84	76	76	85	85	90	90
E4 (3)	95	97	112	112	80	80	101	103	105	105	88	88	97	114	110	119	108	108	86	100	102	102	95	95	86	86	85	85	86	86
E5 (3) *	95	97	112	112	80	100	99	101	120	120	88	133	97	106	119	119	106	108	86	115	102	102	84	112	86	86	75	85	86	90
E6 (3)	97	99	100	112	100	100	101	103	105	105	88	96	114	147	110	119	106	108	86	86	102	102	93	95	76	86	75	85	86	90
E7 (3)	95	97	112	112	80	100	101	103	105	120	88	96	106	114	117	119	106	106	86	115	102	102	84	112	86	86	75	75	90	90
E8 (3)	95	97	112	112	80	100	101	103	105	120	88	96	97	97	119	119	106	108	86	86	102	118	84	93	86	86	75	75	86	90
E9 (5)	97	99	100	112	80	100	101	101	105	120	88	96	97	114	119	119	106	108	86	100	102	102	84	93	76	76	75	75	86	86
E10 (6)	97	97	100	100	80	100	101	101	105	120	96	133	114	147	119	119	106	108	100	100	102	104	84	84	76	86	75	85	86	90
E11 (4)	95	97	112	112	80	100	99	99	105	120	88	96	97	114	117	119	106	108	115	115	102	102	95	112	86	86	75	85	86	90
E12 (7)	97	99	100	100	100	100	101	103	120	120	96	96	106	114	110	119	108	108	100	100	104	118	93	95	76	76	75	85	90	90
E13 (6)	97	99	100	112	80	80	101	101	120	120	88	133	97	114	119	119	106	106	86	100	102	104	84	93	76	76	75	85	86	90
E14 (3)	97	99	112	112	80	100	101	101	105	120	88	96	106	114	117	119	108	108	100	115	102	104	95	95	86	86	75	85	86	90
E15 (3) *	95	97	112	112	100	100	101	103	105	120	88	96	97	97	117	119	108	108	86	115	102	118	93	95	86	86	75	85	86	90

The "alien alleles" (*i.e*., present in none of the 15 breeders) are underlined. Between parentheses after each breeder name is the number of loci that are incompatible with parentage of E1465. The three males in the pool of 15 breeders are indicated with an asterisk.

The most likely explanation is that E1465 is a hybrid between an Española female and a Pinzón male. Inspection of the genotype of E1465 (Table 1) indicates that none of the 12 captive females can be its mother (nor any of the three captive males can be its father), hence, the hybridization event must have taken place on Española (rather than at the captive breeding centre). The cause for the (past or current) presence of a Pinzón male on Española is unknown but is most likely linked to human transport. One possibility is the Pinzón male was a transplant due to the extensive exploitation and sometimes translocation of tortoises by 17–19^th ^century whaling and other activities. It is conceivable that such an animal was missed in the attempts to find all remaining tortoises on Española in 1965. Such transplants have been detected on other islands (e.g., [12]). This would imply Pinzón genetic contamination should have been occurring for several generations, a possibility for which there is no evidence. Indeed, the fact that only a single such hybrid offspring has been found in a sample of 400+ survivors on Española (present data plus those in [9]) is strong evidence against long-term contamination. A more likely scenario is that early in the Española releases (early 1970s), a Pinzón male was accidentally incorporated into the repatriates. A captive breeding program of Pinzón tortoises at the captive breeding centre predates the Española program, so Pinzón juvenile tortoises were present there at the time of release. Furthermore, it is noteworthy that morphologies of Pinzón and Española tortoises, especially as juveniles, are largely indistinguishable.

## Discussions and Conclusion

Clearly, if one wishes to maintain the guideline/principle of restoring "natural" populations [18], it is important to remove from Española the individuals that compromise the integrity of the Española gene pool. In addition to E1465, we also need to search for the possible presence of its father and (half-) siblings. Note that we cannot rule out the possibility that the contaminating Pinzón individual may be a grand-parent of E1465. However, given that our estimate based on size is that E1465 is minimum 9 years old, and tortoises reach sexual maturity at about 20 years of age, it is impossible that E1465 is a grandchild of a male released within the last 35 years. Finding E1465 should be relatively easy because we marked it with a permanent Passive Integrated Transponder tag, as is the case for all individuals that we sampled on the island. Finding the father and its descendants will be more difficult, because they do not carry any tag, as their blood has never been sampled. We will therefore need to search for unmarked tortoises, sample them, and use a diagnostic field-based test that will allow us to differentiate Pinzón *vs*Española alleles. Note that, prior to 1991, all repatriated animals were marked at CDRS using shell notching. Unfortunately, this method is problematic for animals that have grown from about ca. 6 cm to 60–70 cm plastron length in a highly abrasive environment. Hence, we endorse the continued, systematic use of Passive Integrated Transponder tags for marking individuals upon release.

Regardless of the means by which Pinzon alleles have entered the Española lineage, our analysis *(i) *indicates that the rate of contamination of the Española breeding-repatriation program is very low, and *(ii) *underlines the utility of the approach used by the GNP and the CDRS, *i.e*., use molecular genetic approaches to monitor the breeding-repatriation program. One future step might be to routinely perform a diagnostic PCR-based test on all the tortoises that will be released in the future to assess their correspondence with the target population. Despite this detected single contamination, it is highly noteworthy to emphasize the success of this repatriation program conducted by the PNG and CDRS over nearly 40 years in difficult conditions and involving release of over 2000 captive-bred tortoises that now reproduce *in situ*. The recent incorporation of molecular genetic analysis of the program is providing further insights and guidance that will aid in assuring the survival of this unique linage of a spectacular animal.

## Methods

Each sample was collected from the brachial vein of one of the front legs of the tortoise, preserved in a lysis buffer containing 0.1 M of Tris-HCl, 0.1 M of EDTA, 0.2 M of NaCl and 1% sodium dodecyl sulphate (SDS) at a pH of 8.0, and subsequently stored at 4°C. About 200 μl of each blood sample was digested at 37°C overnight in a buffer (100 mM Tris-HCl, 100 mM NaCl, 5 mM EDTA, 0.5% SDS) containing 200 μg of proteinase K. Genomic DNA was isolated following standard phenol-chloroform extraction procedures [19]. DNA was resuspended in Tris-EDTA (TE) buffer (10 mM Tris-HCl and 1 mM EDTA at a pH of 7.2) and stored at -20°C. Microsatellite loci were from those characterized in [9] and in [13]. Genotyping was performed using PCR carried out in a total volume of 25 μl containing 10–100 μg of genomic DNA, 1 × PCR buffer, 2 mM of MgCl2, 0.25 mM of each dNTP, 15 pm of each primer (one with fluorescence labelling, the other with a GTTTCTT tail in 5' to force +A alleles) and 0.7 units of FastTaq DNA polymerase (Roche). Thermal profiles consisted of an initial denaturation step at 95°C for 4 min, followed by 35 cycles of 30 s at 95°C, 30 s at the annealing temperature and 30 s at 72°C, with a final extension step of 60 min at 72°C (to force the formation of +A alleles). PCR products were separated by electrophoresis using an ABI 3730 capillary sequencer. Deterministic and probabilistic parentage analyses were performed with the program PAPA v2.0 [20].

Individuals of unknown ancestry were assigned to island populations based on their multi-locus genotypes using two separate approaches. First, the exclusion-simulation test of a partial Bayesian assignment method [14] was used to assign individuals to their population of origin as implemented in GENECLASS [15]. The exclusion threshold was set to 0.05. In addition, a Bayesian model-based clustering method [16] for inferring population structure and assigning individuals to populations was employed as implemented in STRUCTURE 2.1 [17]. Membership coefficients (*q*) of individuals were estimated following a Markov chain Monte Carlo simulation (MCMC) of 1 × 10^6 ^iterations following an initial burnin of 5 × 10^4 ^repetitions. Given the large body of research directed towards reconstructing population structure and genetic distinctiveness of the extant named taxa of *G. nigra *[12,13,21,22], analyses were run using a model that utilized prior population information, as recommended by [16]. STRUCTURE analysis using a model of population admixture yielded very similar results (data not shown). The mitochondrial control region was sequenced as in [21].

## Authors' contributions

MCM and DM generated the molecular data and performed the data analyses. MCM and JRP wrote the manuscript. MR participated to data analysis. MCM, JG, HLS, WT, CM, and AC localized and sampled all tortoise individuals used in the study. JRP conceived the study. All authors commented on the draft manuscript and approved the final manuscript.
